# Label-free quantitative proteomics of CD133-positive liver cancer stem cells

**DOI:** 10.1186/1477-5956-10-69

**Published:** 2012-11-21

**Authors:** Sheng-Ta Tsai, Chih-Chiang Tsou, Wan-Yu Mao, Wei-Chao Chang, Hsin-Ying Han, Wen-Lian Hsu, Chung-Leung Li, Chia-Ning Shen, Chung-Hsuan Chen

**Affiliations:** 1 Institute of Biochemistry & Molecular Biology, National Yang-Ming University, Taipei, Taiwan; 2 Genomics Research Center, Academia Sinica, Taipei, Taiwan; 3 Institute of Information Science, Academia Sinica, Taipei, Taiwan; 4 Institute of Cellular and Organismic Biology, Academia Sinica, Taipei, Taiwan; 5 Department of Chemistry, National Taiwan University, Taipei, Taiwan; 6 Institute of Atomic & Molecular Sciences, Academia Sinica, Taipei, Taiwan

**Keywords:** Hepatocellular carcinoma, LC-MS/MS, Liver cancer stem cells, Label-free quantitation

## Abstract

**Background:**

CD133-positive liver cancer stem cells, which are characterized by their resistance to conventional chemotherapy and their tumor initiation ability at limited dilutions, have been recognized as a critical target in liver cancer therapeutics. In the current work, we developed a label-free quantitative method to investigate the proteome of CD133-positive liver cancer stem cells for the purpose of identifying unique biomarkers that can be utilized for targeting liver cancer stem cells. Label-free quantitation was performed in combination with ID-based Elution time Alignment by Linear regression Quantitation (IDEAL-Q) and MaxQuant.

**Results:**

Initially, IDEAL-Q analysis revealed that 151 proteins were differentially expressed in the CD133-positive hepatoma cells when compared with CD133-negative cells. We then analyzed these 151 differentially expressed proteins by MaxQuant software and identified 10 significantly up-regulated proteins. The results were further validated by RT-PCR, western blot, flow cytometry or immunofluorescent staining which revealed that prominin-1, annexin A1, annexin A3, transgelin, creatine kinase B, vimentin, and EpCAM were indeed highly expressed in the CD133-positive hepatoma cells.

**Conclusions:**

These findings confirmed that mass spectrometry-based label-free quantitative proteomics can be used to gain insights into liver cancer stem cells.

## Background

Numerous studies have identified a subpopulation of cells in a wide variety of tumors that possess stem cell characteristics, including the ability to self-renew and differentiate into heterogeneous tumor cells [1,2]. These cells are called “cancer stem cells” (CSCs) or “tumor-initiating cells” (TICs) [3]. CSCs were first discovered in leukemia [4,5] and were subsequently identified in various solid tumors, including melanoma [6], breast cancer [7], brain tumors [8,9], prostate cancer [10], head and neck cancer [11], lung cancer [12], colon cancer [13,14], pancreatic adenocarcinoma [15], ovarian cancer [16], and hepatocellular carcinoma (HCC) [17,18]. There is accumulating evidence that CSCs display drug resistance to many conventional therapies which therefore leads to cancer recurrence, suggesting that new cancer therapeutics may be required to target and eliminate the cancer stem cells. Therefore, a better understanding of the mechanisms that control the aspects of self-renewal and survival in cancer stem cells is very important. Furthermore, the identification of unique biomarkers could also facilitate the development of therapeutics that target CSCs.

The CD133 antigen (prominin-1) is a cell-surface glycoprotein that contains 865 amino acids, 5 trans-membrane domains, and 2 glycosylated extracellular loops [19]. The glycosylated CD133 antigen that is recognized by AC133 monoclonal antibodies is a cell surface marker of hematopoietic stem cells and possibly hepatic stem cells [20,21]. Recent findings have also revealed that the glycosylated CD133 antigen is also a potential marker for the isolation of CSCs from a wide variety of tumor tissues, including glioblastomas, lung cancer, pancreatic adenocarcinomas, prostate cancer, colon cancer and hepatocellular carcinomas [8,10,12-14,22-24]. Recent reports suggest a subpopulation of liver cancer cells expressed the glycosylated CD133 antigen are with characteristics of CSCs and can lead to cancer progression and relapse [17,23-25]. More importantly, CD133 is a prognostic marker that has either been linked to poor survival for liver cancer patients [26] or has been correlated with higher grade tumors [27]. Therefore, CD133-positive liver cancer cells have been recognized as a critical target for liver cancer therapies.

The mass spectrometry (MS)-based proteomics technology has been shown as a powerful tool for large-scale protein identification and quantitation. To investigate the differentially expressed proteins in a complex biological system, strategies for reproducible and accurate quantification are required. Several quantitative proteomics methods have been developed, including stable isotope labeling and label-free methods [28]. Although isotope labeling techniques have been widely used in quantitative proteomics research, researchers are increasingly turning to label-free shotgun proteomics techniques. Although both methods have certain strengths and weaknesses, MS-based label-free quantitative proteomics may be adequate for application in complex biological systems [29,30]. Over the past few years, a limited number of groups have applied proteomics to investigate CSCs. For example, Ma et al., and Lee et al. had utilized two-dimension electrophoresis (2-DE) to determine the differently expressed proteins in CD133^+^ liver cancer cells and identified ALDH1A1 and transgelin were highly expressed in CD133^+^ cells compared with CD133^-^ cells [31,32]. Nevertheless, the 2-DE method is less efficient and time-consuming. Recently, Van Houdt et al. applied LC-MS/MS in combination with spectral counting for label-free quantitation to analyze colorectal cancer stem cells and identified BIRC6 as a key mediator of drug resistance against cisplatin and oxaliplatin [33]. However, label-free quantitative proteomics encounters the problem of less accuracy although the method is rather simpler and cheaper.

**Figure 1:**
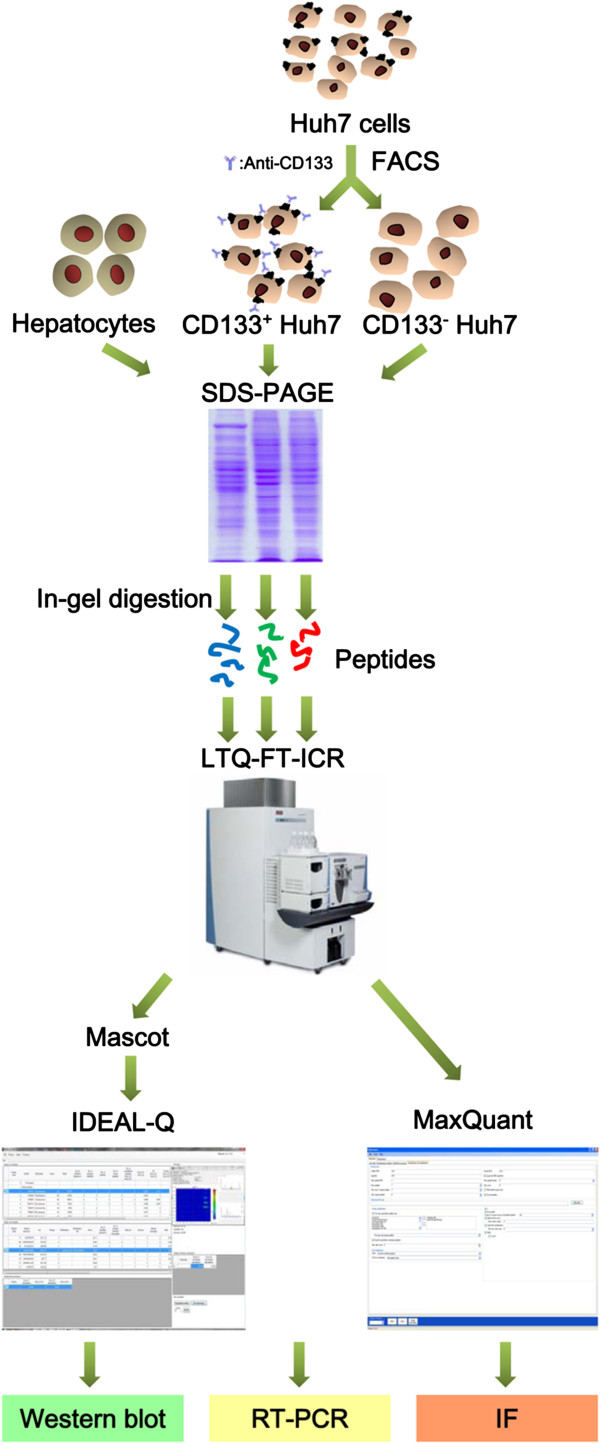
**Experimental scheme for the label-free quantitation analysis of human hepatocytes, CD133**^**+**^**Huh7 cells, and CD133**^**-**^**Huh7 cells.**

In order to improve accuracy of label-free quantitative proteomics, in the current work, label-free quantitation was performed in combination with ID-based Elution time Alignment by Linear regression Quantitation (IDEAL-Q) [34] and MaxQuant [35,36]. Subpopulations of Huh7 hepatoma cells that did or did not express the glycosylated CD133 antigen (prominin-1) were sorted and analyzed by mass spectrometry-based comparative proteomics. The protein abundance in CD133^+/−^ Huh7 hepatoma cells and normal human hepatocytes was initially determined by averaging the peptide abundances with IDEAL-Q which computed the area of the extracted ion chromatogram (XIC) followed with validation by MaxQuant. The experimental design is shown in Figure 1. Using the approach, we established the proteomes of CD133^+/−^ Huh7 hepatoma cells and normal human hepatocytes and identified a number of proteins that differentially expressed in the CD133-positive hepatoma cells. The results showed that the MS-based label-free quantitative proteomics method developed in the current work can be used to gain further insights into liver CSCs.

## Results

### Characterization of the CD133-positive subpopulation of the Huh7 hepatoma cells

**Figure 2:**
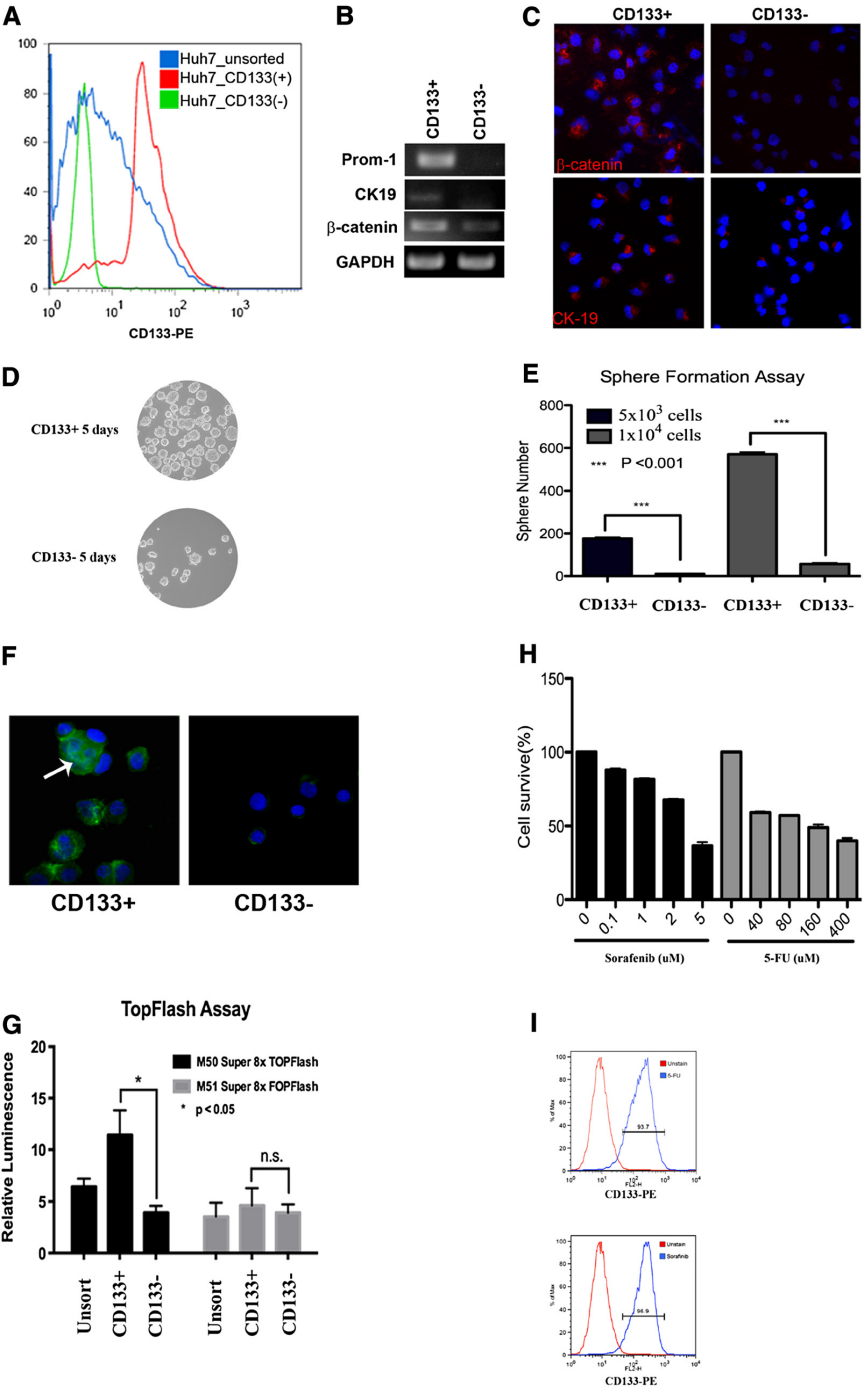
**Characterization of CD133**^**+**^**Huh7 and CD133**^**-**^**Huh7 cells.** (**A**) Re-analysis of FACS-sorted CD133^+^ and CD133^-^ Huh7 cells. (**B**) RT-PCR analysis of CD133^+^ and CD133^-^ Huh7 cells. (**C**) Immunofluorescent staining of CD133^+^ and CD133^-^ Huh7 cells with anti-β-catenin and anti-CK19 antibodies. (**D**) Formation the anchorage-independent spheres in stem cell medium. (**E**) Quantitative analysis of sphere-forming efficiency of CD133^+^ and CD133^-^ Huh7 cells. (**F**) Immunofluorescent staining of CD133^+^ and CD133^-^ Huh7 cells with anti-β-catenin antibody. Arrowed cells showed nuclear staining of β-catenin. (**G**) Huh7 cells were transfected with equal amounts of either Super8xTOPFlash reporter or Super8xFOPFlash reporter constructs. After 48 hours, the TopFlash/FopFlash luciferase activity of CD133^+^ and CD133^-^ Huh7 cells was measured. Values shown are relative luminescence. (**H**) Huh7 cells were treated with different concentration of 5-fluoroucacil (5-FU) or Sorafenib for 48 hours. The cell survival was determined using MTT assay. (**I**) Huh7 cells were treated with 160 μM of 5-FU or 3 μM of Sorafenib for 48 hours. The expression of CD133 in survived cells was analyzed by flow cytometry.

Previous works have demonstrated that Huh7 hepatoma cells contain a CD133-positive subpopulation, which displays the characteristics of cancer stem cells [17,23,25]. We initially determined whether the Huh7 cells contained a subpopulation of CSCs that expressed the CD133 antigen. The Huh7 cell lines were first stained with fluorescence-conjugated primary antibodies against the surface marker CD133 and then analyzed using flow cytometry. We categorized the 20% of cells displaying the strongest fluorescence as CD133^+^ Huh7 cells and the 20% of cells displaying the weakest fluorescence as CD133^-^ Huh7 cells. A re-analysis was performed, which showed that CD133^+^ cells and CD133^-^ cells were indeed two different populations of cells (Figure 2A). Further characterization of the CD133^+^ Huh7 cells by RT-PCR and immunofluorescent staining showed that these cells expressed higher levels of prominin-1, β-catenin, and cytokeratin 19 (CK19) (Figure 2B-C), which confirmed previous findings [17]. In order to determine whether CD133^+^ Huh7 cells expressed characteristics of CSCs, we performed sphere-forming experiments, TopFlash assay and drug resistance test. Indeed, sphere forming assay has been widely used to examine anchorage-independent growth ability of stem cells and CSCs in liver [37-39]. As shown in Figure 2D-E, in comparing with CD133^-^ Huh7 cells, CD133^+^ Huh7 cells generated more spheres. Moreover, Wnt/β-catenin pathway is known to play a key role in the self-renewal of normal and tumorigenic liver stem/progenitor cells [40,41], we then examined if CD133^+^ cells have activated Wnt/β-catenin pathways. Immunofluorescent staining was performed and showed that some CD133^+^ cells had nuclear β-catenin (Figure 2F). TopFlash reporter assay was carried out and further confirmed that CD133^+^ subpopulations had higher Wnt/β-catenin pathway activities compared with CD133^-^ subpopulation or unsorted Huh7 cells (Figure 2G). Since CSCs are known to display drug resistance to many conventional therapies, we then examined the drug resistance of Huh7 cells against two conventional drugs, 5-fluoroucacil (5-FU) and Sorafenib which are widely used in treating HCC. The sensitivity of Huh7 cells was determined using MTT colorimetric assay followed by FACS analysis. As shown in Figure 2H, Huh7 cells displayed resistance to 5-FU or Sorafenib with higher IC50 value of 160 and 3 μM respectively. Most importantly, the majority of survived cells from 5-FU or Sorafenib treatment were CD133^+^ (Figure 2I) suggesting CD133^+^ Huh7 cells display higher drug resistance.

### Determination of the proteomes of the CD133^+^ and CD133^-^ subpopulations of Huh7 cells and normal hepatocytes

**Figure 3:**
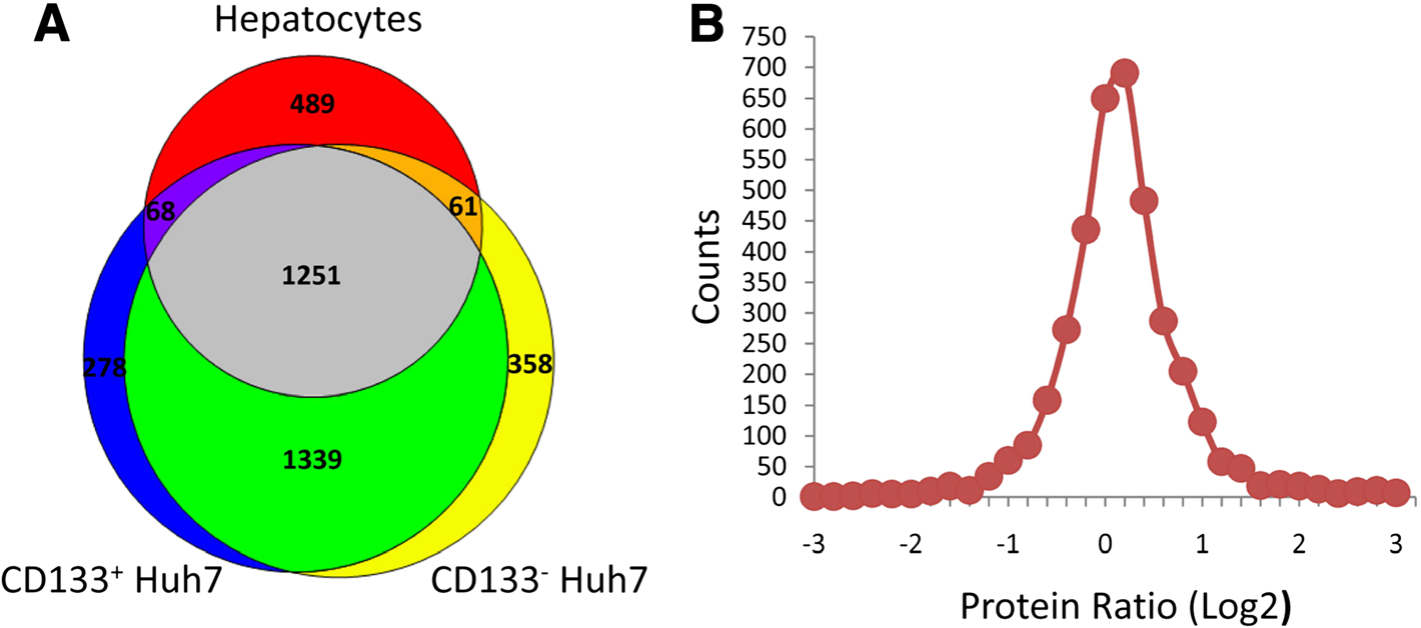
**Proteome analysis.** (**A**) Comparison of the protein profiles of human hepatocytes, CD133^**+**^ Huh7 cells, and CD133^-^ Huh7 cells. The number of identified proteins in human hepatocytes (red), CD133^+^ Huh7 cells (blue), and CD133^-^ Huh7 cells (yellow) are shown by a Venn diagram. The other colors represent the overlap regions. (**B**) The protein ratio distribution of CD133^+^ Huh7 cells/CD133^-^ Huh7 cells.

To identify the differentially expressed proteins in CD133^+^ liver cancer stem cells, we determined the proteomes of human hepatocytes and the CD133^+^ and CD133^-^ subpopulations of the Huh7 cells. All protein extracts, isolated from either the hepatocytes or the CD133^+^ and CD133^-^ subpopulations, were analyzed twice using LC LTQ-FT-ICR. We identified 13,643, 17,707, and 21,865 non-redundant peptides from extracts of the human hepatocytes, CD133^+^, and CD133^-^ Huh7 subpopulations, respectively. Additionally, 1,869, 2,936, and 3,009 proteins were identified from extracts of the human hepatocytes, CD133^+^, and CD133^-^ subpopulations, respectively. Among the proteins identified, 1,471, 2,260, and 2,567 proteins, respectively, had more than 2 peptides that were detected. A comparison of the proteins identified in the three cell types is shown in Figure 3A. The detailed information on the complete proteomes of the human hepatocytes, CD133^+^ Huh7 cells, and CD133^-^ Huh7 cells are included in Additional file 1: Table S1, Additional file 2: Table S2 and Additional file 3: Table S3.

### Identification of the significantly up-regulated and down-regulated proteins in the CD133^+^ subpopulations of the Huh7 cells

**Figure 4:**
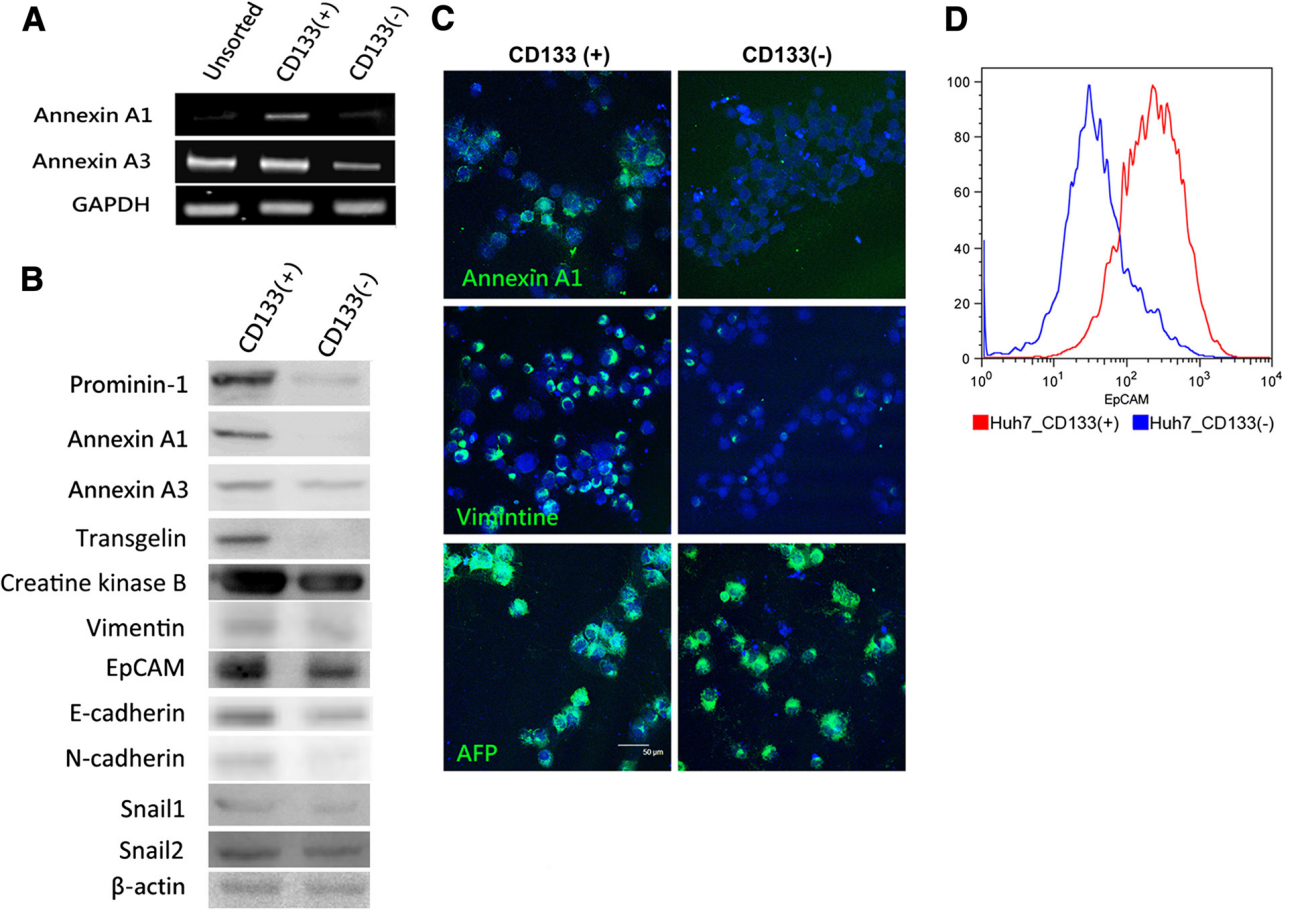
**Validation of significantly up-regulated proteins in CD133**^**+**^**Huh7 and CD133**^**-**^**Huh7 cells.** (**A**) RT-PCR, (**B**) western blot analysis, (**C**) immunofluorescent staining, and (**D**) FACS analysis were performed to validate significantly up-regulated proteins in CD133^+^ and CD133^-^ Huh7 cells. GAPDH in the RT-PCR and β-actin in the western blot were used as loading controls.

To identify proteins that were either significantly up-regulated or down-regulated in the CD133^+^ subpopulation, IDEAL-Q was used to calculate the ratios of the protein abundance and to determine the significant difference (*p*<0.01) in the ratios of the protein abundance between the CD133^+^ and CD133^-^ subpopulations. The distribution of the ratios of the protein abundance between the CD133^+^ and CD133^-^ subpopulations is shown in Figure 3B. The data showed no significant difference in the level of most of the proteins, and a total 151 proteins were found to be differentially expressed in the CD133^+^ subpopulations when compared with the CD133^-^ subpopulations. In order to select the specific target proteins in the CD133^+^ subpopulations, the differentially expressed proteins were also compared with those in normal human hepatocytes and further analyzed by MaxQuant [35]. Based on the IDEAL-Q and MaxQuant analysis, we found 10 proteins that were abundantly expressed in the CD133^+^ cell populations when compared to the CD133^-^ cell populations and the normal human hepatocytes (Table 1). These proteins have been quantified by using more than three unique peptides. The difference in the expression level of these 10 proteins in CD133^+^, CD133^-^ Huh7 cell populations, and the normal human hepatocytes was statistically significant. Since, the subpopulations of the Huh7 cells were sorted based on the expression of prominin-1 (CD133), and the analytic data showed the amount of prominin-1 protein in the CD133^+^ Huh7 cells was approximately 15-fold higher than that in the CD133^-^ Huh7 cells and 76-fold higher than that in the human hepatocytes; hence, these results suggest that the quantitative analysis using IDEAL-Q is reliable. As expected, several markers of liver cancer stem cells or hepatic stem cells could also been identified by IDEAL-Q and shown to be up-regulated in the CD133^+^ subpopulations. For example, transgelin, an actin-binding protein, has been proven to be highly expressed in CD133^+^ Huh7 cells [31]. In our study, the expression level of transgelin was 16-fold higher in the CD133^+^ subpopulations than that in the CD133^-^ populations. Among the proteins that were significantly up-regulated in the CD133^+^ subpopulations of the Huh7 cells, we verified that the CD133^+^ Huh7 cells expressed higher levels of annexin A1, annexin A3, prominin-1, transgelin, creatine kinase B, vimentin, and EpCAM (Figure 4A-D), using RT-PCR, western blot, immunocytochemical analysis, and FACS analysis. The level of epithelial cell adhesion molecule (EpCAM), which has also been shown to be highly expressed in liver cancer stem cells [42], was 2.6-fold higher in the CD133^+^ subpopulations compared with the CD133^-^ subpopulations. The expression level of EpCAM in CD133^+^ and CD133^-^ populations were also detected by flow cytometry (Figure 4D). Two other hepatic stem cell markers, vimentin and cytokeratin 19, were 8-fold and 3.7-fold higher, respectively, in the CD133^+^ subpopulations when compared to the CD133^-^ subpopulations. Since it has been shown that Snail1-induced epithelial-to-mesenchymal transition is accompanied by enhancement in stem cell characteristics during liver carcinogenesis [43], we also examined the level of mesenchymal markers by western blot (Figure 4B). Although the level of E-cadherin was not decreased in CD133^+^ Huh7 cells, however, in addition to vimentin, up-regulation of N-cadherin was also observed. Taken together, these results suggest that mass spectrometry-based label-free quantitative proteomics developed in the current study can be used to gain the insights into liver cancer stem cells.

**Table 1:** **Significantly up-regulated proteins in the CD133**^**+**^**Huh7 cells**

**Accession**	**Protein description**	**Score**	**Unique peptides**	**Hepatocyte area**	**CD133**^**-**^**Huh7 area**	**CD133**^**+**^**Huh7 area**	**IDEAL-Q CD133**^**+**^**/CD133**^**-**^	**IDEAL-Q CD133**^**+**^**/Hepatocyte**	**P-value**^**a**^	**MaxQuant CD133**^**+**^**/CD133**^**-b**^	**Plasma membrane**^**c**^
IPI00216138	Transgelin	552	10	0.21 ± 0.05	0.13 ± 0.06	2.11 ± 0.30	15.96	9.99	<0.001	33.94	
IPI00012540	Prominin-1 (CD133)	170	4	0.01 ± 0.01	0.05 ± 0.01	0.69 ± 0.09	15.24	76.22	<0.001	124.06	O
IPI00418471	Vimentin	1726	30	0.38 ± 0.03	0.82 ± 0.06	6.77 ± 0.47	8.24	17.77	<0.001	21.51	O
IPI00218918	Annexin A1	303	5	0.02 ± 0.002	0.04 ± 0.01	0.33 ± 0.03	7.83	21.93	<0.001	101.44	O
IPI00186460	Collagen alpha-1(II) chain	227	4	0.01 ± 0.01	0.15 ± 0.05	0.94 ± 0.06	6.10	67.07	<0.001	11.95	
IPI00024095	Annexin A3	919	15	0.15 ± 0.003	0.44 ± 0.07	2.69 ± 0.48	6.10	17.70	<0.001	17.13	O
IPI00329650	Nucleoporin NUP53	252	4	0.05 ± 0.02	0.28 ± 0.04	1.29 ± 0.13	4.71	26.41	<0.001	3.62	O
IPI00296215	Tumor-associated calcium signal transducer 1 (EpCAM)	204	3	0.01 ± 0.004	0.12 ± 0.01	0.31 ± 0.02	2.56	27.73	0.010	6.17	O
IPI00386208	Gastric-associated differentially-expressed protein YA61P	224	4	0.01 ± 0.01	0.42 ± 0.04	0.97 ± 0.07	2.34	108.22	0.008	13.90	
IPI00022977	Creatine kinase B-type	1181	19	0.86 ± 0.15	6.39 ± 0.58	13.50 ± 2.43	2.11	15.66	0.009	2.03	

^a^ Significance B based on log(2) transformed ratios and intensity between CD133^+^ Huh7 cells and CD133^-^ Huh7 cells.

^b^ The ratio was calculated by MaxQuant label-free quantification.

^c^ Proteins possibly locate on plasma membrane from Gene Ontology Cellular Component (GOCC) database.

In addition to the significantly up-regulated proteins, we also found 60 proteins that were down-regulated in the CD133^+^ subpopulations when compared to the CD133^-^ subpopulations. The detailed list of the up- and down-regulated proteins is listed in Additional file 4: Table S4. Furthermore, in order to investigate the protein locations of the identified proteins, the Gene Ontology cellular component (GOCC) terms were annotated for the identified proteins. Twenty-one plasma membrane proteins had higher expression levels in the CD133^+^ Huh7 cells than in the CD133^-^ Huh7 cells, six of them which has been listed in Table 1 had higher expression levels (>10-fold) in the CD133^+^ Huh7 cells when compared with the hepatocytes. We therefore proposed that these up-regulated plasma membrane proteins can possibly be used as markers for the enriched isolation of liver cancer stem cells.

### Signaling molecules identified in the CD133^+^ Huh7 cells

**Figure 5:**
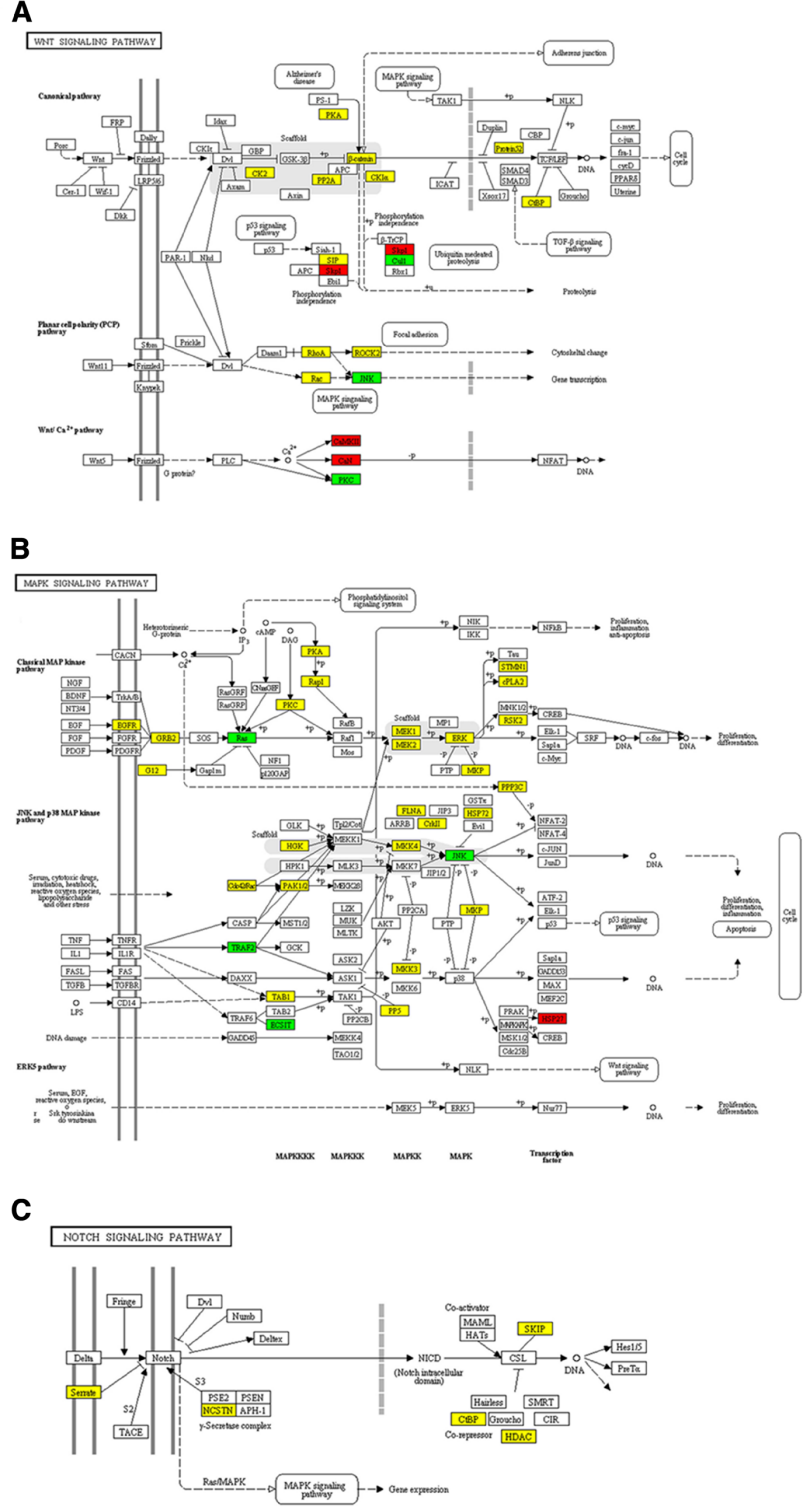
**Pathway analysis.** Proteins involved in (**A**) Wnt/β-catenin pathway, (**B**) MAPK pathway, and (**C**) Notch pathway. The identified proteins were up-regulated (red), down-regulated (green), or showed no significant change (yellow) in CD133^+^ Huh7 cells.

Several signaling pathways have been found in liver cancer stem cells that are involved in self-renewal, survival signaling, and drug resistance [25,42]. For example, activation of the Wnt/β-catenin pathway has been shown to be associated with a wide variety of cancers [44] and it plays a very important role in regulating the self-renewal of various stem cells and liver progenitors [40]. To investigate the signaling pathway that governing the behavior of liver cancer stem cells, the Kyoto Encyclopedia of Genes and Genomes (KEGG) terms were annotated for the identified proteins. As expected, 21 of the identified proteins were involved in the Wnt/β-catenin pathway. In addition to Wnt/β-catenin pathway, 41 proteins involved in the MAPK pathway, and 6 proteins involved in the Notch signaling pathway have also been identified (Figure 5). The results implied both MAPK and Notch signaling pathways would also be critical for regulating the tumorigenic behavior of liver CD133^+^ CSC subpopulations.

**Figure 6:**
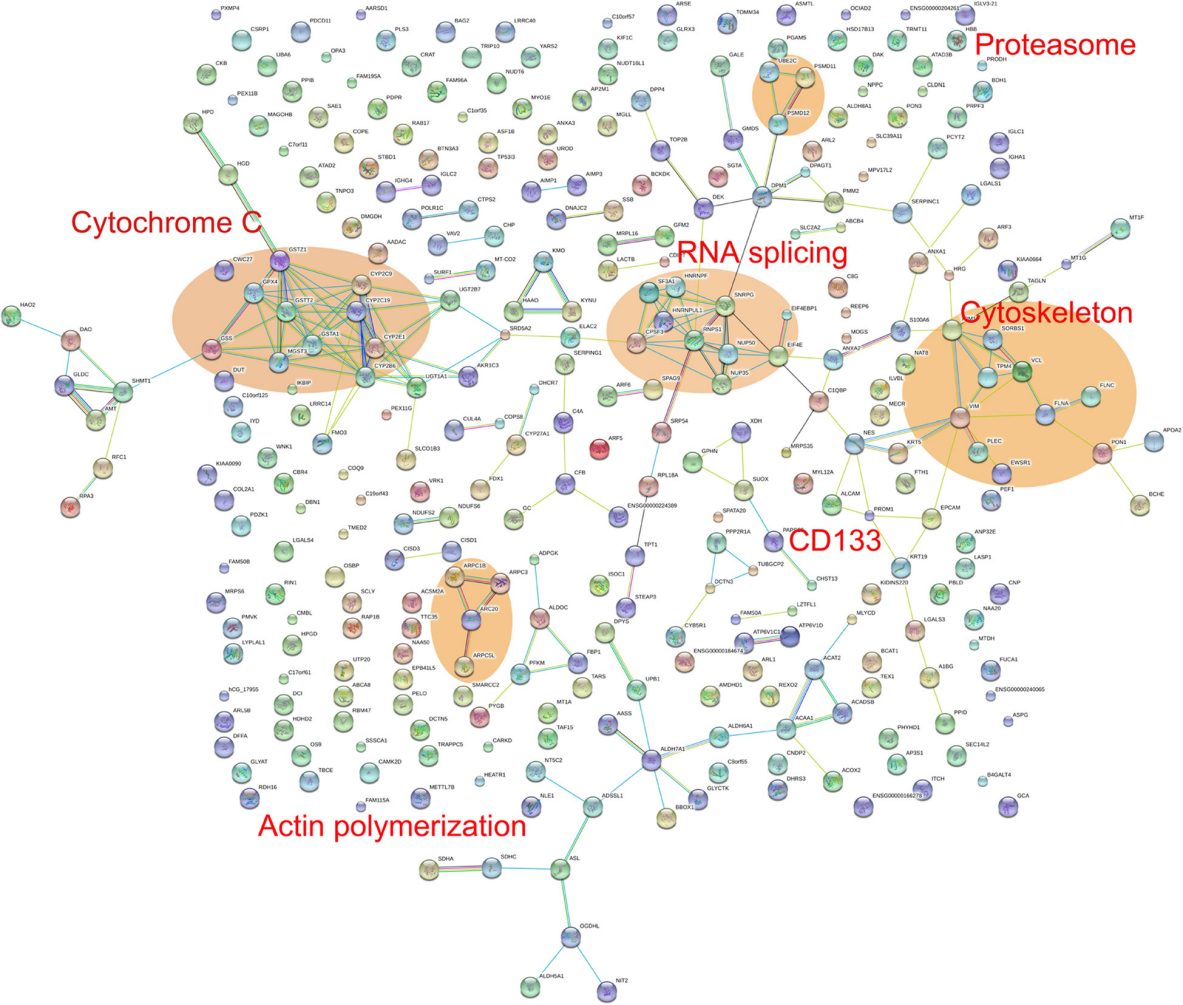
String database mapping reveals the interaction groups of the significantly regulated proteins.

To investigate the protein-protein interactions, the proteins were mapped with the Search Tool for the Retrieval of Interacting Genes/Proteins (STRING) database version 9. STRING is constructed based on both physical and functional interactions. The differentially expressed 151 proteins were analyzed by STRING. To avoid spurious interactions in our large data set, we fetched all interactions that had a confidence score of ≧0.7 (high confidence). Several interaction groups were immediately apparent and these interaction groups were labeled orange circles (Figure 6). Prominin-1 was correlated with CK-19, nestin, and EpCAM. These proteins were also found to interact with vimentin. Vimentin is a type III intermediate filament (IF) protein that is expressed in liver stem cells. We also found groups of interaction proteins that were involved in the regulation of cytochrome c, glutathione, RNA splicing, and actin polymerization.

## Discussion

In the current study, we not only identified the proteomes of human hepatocytes and CD133^+/−^ Huh7 cells, but we also quantified the abundance of each protein and presented them in parts per million (ppm), which was then used to determine the protein concentration and to classify the individual protein as either a major or minor component. In addition, the protein ratio was used to determine the relative abundance in the CD133^+/−^ subpopulation. We found that there were 2,590 proteins (77%) detected in both the CD133^+^ and CD133^-^ Huh7 cells, but only 1,251 proteins (32%) were detected in all three cell types, suggesting the CD133^+^ cells and CD133^-^ Huh7 cells have similar characteristics. Some of the calculated protein ratios were found to be different between the IDEA-Q results and the MaxQuant results. Both IDEA-Q and MaxQuant utilized the peptide intensity, retention time and mass to charge, but IDEAL-Q calculated the top three most abundant peptides and determined the protein ratio by dividing the average value. In contrast, the MaxQuant platform calculated the protein ratios as the median of all the ratios for the common peptides. The same method was also applied on a second liver cancer cell line, PLC/PRF/5 that expressed the hepatitis B surface antigen (HBsAg). We confirmed that annexin A1, annexin A3, creatine kinase B, and transgelin were also up-regulated in CD133^+^ PLC/PRF/5 cells. The proteomics results of PLC/PRF/5 cells are listed in Additional file 5: Table S5). We presume that this difference could be due to heterogeneity and the different causes of liver cancer. The identified proteins were searched against the GOCC, and we classified 409 IDs as plasma membrane proteins, including hepatic stem cell markers (CD133, CK19, EpCAM, and vimentin). These up-regulated plasma membrane proteins can be used as new surface targets to enrich or investigate liver CSCs. Interestingly, annexin A1 and annexin A3 were both up-regulated in the CD133^+^ Huh7 cells (7.8- and 6.1-fold, respectively). We found that annexin A2 was also slightly up-regulated (1.9-fold). These three proteins all belong to the same subgroup of the annexin family. Annexin A1 binds acidic phospholipids with a high affinity in the presence of calcium ions. It is also an endogenous anti-inflammatory protein that has roles in many diverse cellular functions, such as membrane aggregation, inflammation, phagocytosis, proliferation, and apoptosis [45]. In a previous study, primary hepatocytes isolated from normal mice did not express annexin A1, but it was strongly expressed in the liver cells of transgenic mice during the development of hepatocarcinomas. Annexin A1 can be expressed by stimulating epidermal growth factor (EGF) or hepatic growth factor (HGF) [46]. In addition, the up-regulation of annexin A1 in hepatic carcinogenesis has been correlated with the activation of the EGF pathway [47]. In this study, we also observed more protein expression of EGF in the CD133^+^ Huh7 cells. Therefore, annexin A1 could be a potential marker for targeting liver CSCs. Annexin A3 had an approximately six-fold higher expression level in the CD133^+^ Huh7 cells compared with the CD133^-^ Huh7 cells. The role of annexin A3 in cancer is still not fully understood, however, some proteomic studies have shown that annexin A3 is a biomarker in lung adenocarcinomas [48], and an overexpression of annexin A3 was detected in platinum-resistant human ovarian cancer cells [49,50]. In the human prostate cancer xenograft model, annexin A3 was up-regulated and drug-resistant both *in vivo* and in the xenograft [51]. In a previous study, annexin A3 was activated by a hepatocyte growth factor pathway and played an important role in rat liver regeneration [52]. This implies that annexin A3 may be activated by a hepatocyte growth factor in the CD133^+^ Huh7 cells and is involved in liver tumor growth. In this study, EpCAM was highly expressed in the CD133^+^ Huh7 cells, and it was found to interact with CD133. EpCAM is a biomarker for hepatic stem cells, and it is also expressed in embryonic stem cells [53-56]. In 2009, EpCAM^+^ HCC cells were identified as possible liver cancer stem cells, and the expression of EpCAM is regulated by Wnt/β-catenin signaling [42]. Currently, several antibody-based therapeutic approaches targeting EpCAM are being developed [57,58]. These reports suggest that EpCAM is not only a biomarker of liver cancer stem cells but also may be a therapeutic target. Metastasis is the main cause of lethality in cancer patients. Cancer stem cells are responsible for both tumor invasion and metastasis [7,18]. In the metastasis process, epithelial-mesenchymal transition (EMT) is a transient and reversible switch from an epithelial to a mesenchymal cellular phenotype, to become highly motile and invasive. EMT is regulated by the Wnt/β-catenin, TGFβ, and Notch pathways. In this study, we found several proteins that are involved in EMT and that were also up-regulated in the CD133^+^ Huh7 cells, such as transgelin, vimentin and collagen. Transgelin is a target of TGFβ signaling that regulates migration and invasion [59]. In addition, it is also co-expressed with several EMT-associated genes, including N-cadherin, vimentin, Snail, and Twist [31]. Vimentin is a mesenchymal marker that was also up-regulated in the CD133^+^ Huh7 cells. Vimentin was also found to be over-expressed in the HCC tissues, and it is involved in the metastasis of HCC [60]. In addition, vimentin has been found to be expressed in multipotent progenitor cells from human fetal livers [53]. Therefore, we identified higher levels of vimentin in CD133^+^ Huh7 cells, which may imply that the expression of vimentin is an important characteristic of liver CSCs. Translationally controlled tumor protein (TCTP) is a highly conserved, hydrophilic nuclear protein. TCTP is involved in many cellular processes [61,62]. For example, TCTP interacts with BCL-X_L_ to protect cells against apoptosis [63,64]. More importantly, a recent report has shown that TCTP is a transcription factor that regulates the pluripotent gene *oct4*[65], which is the key factor in regulating the pluripotency of embryonic stem cells. Thus, the higher levels of TCTP identified in the CD133^+^ Huh7 cells may imply that TCTP plays a role in regulating the stemness properties of liver CSCs.

## Conclusions

With the assistance of the label-free quantitation analysis tool IDEAL-Q and MaxQuant, the current work identified several candidate proteins of liver cancer stem cells, which can possibly be used in the future for targeting liver cancer stem cells. Moreover, the proteome analysis utilized in the present work has provided a comprehensive characterization of CD133^+^ liver cancer stem cells, CD133^-^ liver cancer cells, and human hepatocytes. We believe the proteome established in the current work can aid in the understanding of the molecular basis of the tumorigenic liver CSC subpopulations.

## Methods

### Cell culture

The hepatoma cell lines Huh7 and PLC/PRF/5 [66] were maintained in Dulbecco’s modified Eagle’s medium (Hyclone, South Logan, UT, USA) supplemented with 10% fetal bovine serum (FBS) (Invitrogen, Carlsbad, CA, USA), 100 units/ml of penicillin, and 100 μg/ml of streptomycin (Invitrogen) at 37°C in a humidified atmosphere containing 5% CO_2_. Human hepatocytes, purchased from PromoCell, were maintained in Hepatocyte Growth Medium (PromoCell, HD, GRE) at 37°C in a humidified atmosphere containing 5% CO_2_.

### Fluorescence-activated cell sorting

To isolate the CD133^+^ and CD133^-^ fractions, Huh7 cells were re-suspended in Hank’s balanced saline solution (HBSS; Invitrogen) containing 2% FBS and 10 mM HEPES. The cell density was adjusted to 1 × 10^7^/ml, and the Huh7 cells were stained with phycoerythrin (PE)-conjugated anti-human CD133 antibody (Miltenyi Biotec Inc., Cambridge, MA, USA). After staining, the Huh7 cells were re-suspended in HBSS containing 2% FBS and 1 mM HEPES and filtered through a 40 μm mesh filter, and the CD133^+^ and CD133^-^ Huh7 fractions were sorted by a FACSAria™ (Becton Dickinson, San Jose, CA, USA).

### Sphere forming assay

Freshly FACS-sorted CD133^+^ and CD133^-^ Huh7 were plated into ultra-low attachment 6-well plates (Costar, Coining Inc) and cultured in DMEM/F12 medium supplemented with 1X B27 supplement, 1% penicillin-streptomycin, 0.4% bovine serum albumin, 200 ng/mL epidermal growth factor (EGF), 10 ng/mL basic fibroblast growth factor (bFGF), 5 μg/mL insulin at 37°C in a humidified atmosphere containing 5% CO_2_. Spheres were formed after 24 hours. The number of spheres that had diameter over 100 μM was count at Day 5.

### RNA extraction and RT-PCR

To isolate the total RNA, 1 × 10^6^ cells of both the CD133^+^ and CD133^-^ Huh7 cell fractions were collected in microcentrifuge tubes containing 1 ml TRI Reagent® (Applied Biosystems, Foster City, CA, USA). The RNA was extracted according to the manufacturer’s protocol and treated with RQ-1 DNase (Promega, Madison, WI, USA) to remove contaminating genomic DNA. The purity and concentration of the RNA were determined with a NanoDrop® (NanoDrop Technologies, Wilmington, DE, USA). First strand complementary DNA was synthesized using SuperScript III reverse transcriptase (Invitrogen). The PCR reactions were performed with annealing temperatures of 57-58°C for either 23 or 35 cycles. The primer sequences used are listed in Additional file 6: Table S6).

### Immunofluorescence staining

The Huh7 cells were either cultured on non-coated glass coverslips or cytospined onto slides at 250 × *g* for 5 minutes. The cells were fixed with 4% paraformaldehyde in PBS for 30 minutes, permeabilized with 0.1% (v/v) Triton X-100 in PBS for 30 minutes, and incubated in 2% blocking buffer (Roche, Indianapolis, IN, USA) before sequential incubation with the primary and secondary antibodies. The antibodies were obtained as follows: mouse anti-human β-catenin was obtained from Becton Dickinson, rabbit anti-human α-fetoprotein was obtained from Dako, mouse anti-cytokeratin 19 and mouse anti-vimentin were purchased from Sigma, and the annexin A1 antibody was purchased from Abnova (Taipei City, Taiwan).

### Western blotting

The cell extracts were prepared by lysing unsorted, CD133^+^, or CD133^-^ Huh7 cells with RIPA buffer containing 150 mM NaCl, 50 mM Tris–HCl (pH 8), 1% NP-40, 0.5% sodium deoxycholate, 0.1% SDS, protease inhibitors, and phosphatase inhibitors (Sigma). The cell extracts were run on an 8-10% SDS-PAGE gel and transferred onto a Hybond-P membrane (GE Healthcare, Buckinghamshire, NA, England), which was then probed with the primary antibodies. The antibodies used in the experiments are listed in Additional file 7: Table S7). The signal was detected with an enhanced chemiluminescence kit (GE Healthcare).

### TopFlash assay

TOPFlash reporter (containing seven TCF/LEF binding sites) and FOPFlash reporter (containing six mutant TCF/LEF binding sites which was used as a negative control.) plasmids were originally obtained from Addgene (Dr. Randall Moon). Huh7 cells plated in 6-well plates at the density of 3 × 10^5^ cells/well were grown for 24 hours. Either TOPFlash or FOPFlash reporter plasmids (1 μg/well) was transfected together with Renilla control luciferase plasmid (100 ng/well) using Fugene HD (Roche) according to the manufacturer’s instructions. 48 hours after transfection, Huh7 cells were harvested and stained with PE conjugated anti-human CD133 antibody. The CD133^+^ and CD133^-^ Huh7 fractions were sorted by a FACSAria. After sorting, CD133^+^ and CD133^-^ Huh7 cells were lysed, and then firefly and Renilla luciferase activities were measured using the Dual-Luciferase Reporter Assay System (Promega).

### Drug resistance assay

Huh7 cells were seeded at the density of 7,500 cells/well in 96-well plates and were treated with a serial dilution of 5-FU or Sorafenib for 48 hours. The cytotoxicity of the drug was determined using a 3-(4,5-dimethylthiazol-2-yl)-2,5-diphenyltertrazolium bromide (MTT) colorimetric assay (Sigma).

### In-gel digestion

The cell lysates were separated on a 12% polyacrylamide gel (10 × 10 cm, 0.75 mm thick), and the protein patterns were observed by Coomassie blue staining. Each lane was separated into 16 sections, and each section was cut into 0.75 mm^3^ cubes. The gel pieces were de-stained with a 25 mM NH_4_HCO_3_/50% acetonitrile (ACN) solution. After reduction and alkylation reactions using 10 mM DTT and 55 mM iodoacetamide, respectively, the proteins were digested overnight with modified trypsin (Promega, Madison, WI, USA) at 37°C. The tryptic peptides were extracted with 1% trifluoroacetic acid (TFA)/60% ACN. After lyophilization, the extracted peptides were re-dissolved in a 0.1% TFA solution.

### LC-MS/MS analysis

The mass analysis experiments were performed using a 7-Tesla LTQ-FT-ICR (Linear quadrupole ion trap Fourier transform ion cyclotron resonance) mass spectrometer (Thermo Fisher Scientific, Waltham, MA, USA) equipped with a nanoelectrospray ion source (New Objective Inc., Woburn, MA, USA), an Agilent 1100 Series binary high-performance liquid chromatography pump (Agilent Technologies, Palo Alto, CA, USA), and a FAMOS autosampler (LC Packings, San Francisco, CA, USA). The peptide mixtures were injected (5 μl) at a flow rate of 10 μl/min onto a self-packed pre-column (150 μm I.D. × 15 mm, 5 μm, 100 Å). The chromatographic separation was performed on a self-packed reversed phase C18 nano-column (75 μm I.D. × 300 mm, 5 μm, 100 Å) at 300 nl/min with a 60 min gradient of 0 to 32% acetonitrile in 0.1% formic acid. The electrospray voltage was maintained at 2.5 kV, and the capillary temperature was set at 200°C. The full-scan MS spectra (*m/z* 300–1800) were acquired with the FT-ICR with a mass resolution of 100,000 at *m/z* 400. After the completion of the full-scan survey, the 10 most abundant ions detected in the full-MS scan were selected for peptide sequence determinations using MS/MS experiments. Each sample was analyzed in duplicate.

### Protein identification

The peak-list files were obtained from the MS/MS data using Extract_msn 4.0 software (Thermo), which included the mass values and the intensity and charge of the precursor ions (parent ions with +2 or +3 charges in this study). For protein identification, the MS/MS data were submitted to the International Protein Index (IPI) human protein database (release 3.43, 72,340 sequences) using the MASCOT 2.2.2 search engine (Matrix Science, Boston, MA, USA) with the following settings: trypsin cleavage; a peptide mass matching error tolerance of 5 ppm; a fragment mass tolerance of 0.5 Da; fixed modification of carbamidomethylation of cysteine; variable modifications of deamidation of asparagine and glutamine, oxidation of methionine; a maximum of two missed cleavages; an ion score cutoff of 33 (*p* < 0.003); and a false discovery rate of < 1.1%. The false discovery rate was calculated by decoy database searching. The results were imported into Microsoft Excel for further analysis.

### Quantitative data analysis

The IDEAL-Q method, which was developed previously [34], was utilized to process the LC-MS/MS data, and the Mascot search engine was used to extract the quantitative information. In brief, the raw data files were first converted into the mzXML data format by the ReAdW program (http://tools.proteomecenter.org/wiki/index.php?title=Software:ReAdW). The resulting mzXML files and the Mascot search results for the peptide and protein identification were used as the input for the quantitative tool. To process a peptide of an LC-MS run, we extracted the LC-MS data within the range of ± 1.5 minutes of its elution time and ± 3.5 Da of the precursor *m/z* value for quantitative analysis. We used the extracted ion chromatogram (XIC) to determine the peptide abundance in an LC-MS run. After determining the peptide abundance for each LC-MS run, we calculated the fraction’s peptide abundance by averaging the peptide abundances of the valid peptides in all of the LC-MS runs for the fraction. To determine the peptide abundance in a sample, we summed all of the peptide fraction abundances in the sample, and the peptide ratio between two samples was also calculated. To calculate the protein ratio, we selected the non-degenerate peptides and performed the Dixon’s test to eliminate the outliers for each protein. We then averaged the abundances of the top three most abundant peptides, and the protein ratio was determined by dividing the average value for each of the different cell types. The up-regulated or down-regulated proteins were defined as having a protein ratio with a significant change (*p *< 0.01). Label-free quantitation was also performed in MaxQuant [35,36] version 1.2.0.18. The significance B and p-values were calculated by Perseus software.

## Abbreviations

ACN: Acetonitrile; CSCs: Cancer stem cells; HCC: Hepatocellular carcinoma; IDEAL-Q: ID-based Elution time alignment by linear regression quantitation; PE: Phycoerythrin; TICs: Tumor-initiating cells; XIC: Extracted ion chromatogram.

## Competing interests

The authors declare that they have no competing interests.

## Authors’ contributions

STT carried out the experiment, analyzed data and wrote the manuscript. CCT and WLH created the software to analyzed data. WYM, HYH collected the cells and participated in the experiments. WCC provided expertise in experimental design and interpretation of analytical results. CLL help to develop Topflash assay. CNS and CHC conceived of the study, interpretation of analytical results and drafted the manuscript. All authors read and approved the final manuscript.

## Supplementary Material

Additional file 1: Table S1 The information of identified proteins in hepatocytes. Click here for file

Additional file 2: Table S2 The information of identified proteins in CD133^**+**^ Huh7 cells. Click here for file

Additional file 3: Table S3 The information of identified proteins in CD133^**-**^ Huh7 cells. Click here for file

Additional file 4: Table S4 Significant proteins in CD133^**+**^ Huh7 cells. Click here for file

Additional file 5: Table S5 The information of identified proteins in PLC/PRF/5 cells. Click here for file

Additional file 6: Table S6 The primer sequences for RT-PCR. Click here for file

Additional file 7: Table S7 The antibodies information for Western blot. Click here for file
